# Quantifying the interconnectedness between poverty, health access, and rabies mortality

**DOI:** 10.1371/journal.pntd.0011204

**Published:** 2023-04-20

**Authors:** Emma Taylor, Katy George, Emily Johnson, Hannah Whitelegg, Joaquin M. Prada, Daniel L. Horton

**Affiliations:** Department of Comparative Biomedical Sciences, School of Veterinary Medicine, University of Surrey, Guildford, United Kingdom; Tufts Medical Center, UNITED STATES

## Abstract

The global 2030 goal set by the World Organization for Animal Health (WOAH), the World Health Organization (WHO), and the Food and Agriculture Organization (FAO), to eliminate dog-mediated human rabies deaths, has undeniably been a catalyst for many countries to re-assess existing dog rabies control programmes. Additionally, the 2030 agenda for Sustainable Development includes a blueprint for global targets which will benefit both people and secure the health of the planet. Rabies is acknowledged as a disease of poverty, but the connections between economic development and rabies control and elimination are poorly quantified yet, critical evidence for planning and prioritisation. We have developed multiple generalised linear models, to model the relationship between health care access, poverty, and death rate as a result of rabies, with separate indicators that can be used at country-level; total Gross Domestic Product (GDP), and current health expenditure as a percentage of the total gross domestic product (% GDP) as an indicator of economic growth; and a metric of poverty assessing the extent and intensity of deprivation experienced at the individual level (Multidimensional Poverty Index, MPI). Notably there was no detectable relationship between GDP or current health expenditure (% GDP) and death rate from rabies. However, MPI showed statistically significant relationships with per capita rabies deaths and the probability of receiving lifesaving post exposure prophylaxis. We highlight that those most at risk of not being treated, and dying due to rabies, live in communities experiencing health care inequalities, readily measured through poverty indicators. These data demonstrate that economic growth alone, may not be enough to meet the 2030 goal. Indeed, other strategies such as targeting vulnerable populations and responsible pet ownership are also needed in addition to economic investment.

## Author summary

The 2030 agenda for Sustainable Development, details global targets to ensure improvements to health and well-being globally. SDG states that to meet SDG 1 and 3, poverty everywhere must end, and universal health coverage and access to quality health care must be achieved. Rabies is acknowledged as a disease of poverty, and therefore ending poverty, and improving health and economic deprivation, could in theory result in a reduction of rabies, providing it also results in universal health coverage. We investigated whether rabies incidence is an inevitable consequence of poverty or whether measures of development other than economics, are stronger predictors of countries that have successfully controlled the disease. We highlight that those most at risk of not being treated, and dying due to rabies, live in communities experiencing health care inequalities, readily measured through poverty indicators. We found that economic growth alone, may not be enough to support the global 2030 goal of zero human death due to dog rabies. Indeed, other strategies such as targeting vulnerable populations, responsible pet ownership are also needed in addition to economic investment.

## 1. Introduction

Progress has been made in the battle against Neglected Tropical Diseases (NTDs), which has helped to alleviate the health and economic burden and resulted in recognition that NTDs are important indicators for the health of communities [1,2]. The new NTD roadmap identifies existing gaps and critical actions needed to reach the 2030 targets and defines global targets which, if achieved, will help to prevent, control and eliminate the 20 recognised diseases and disease groups [3,4]. Rabies is one of the 20 recognised diseases, with a target of elimination as a public health problem, with the global goal of zero human deaths as a result of dog-mediated rabies [5]. The number of countries having achieved zero human deaths from rabies is projected to increase from 80 (47%) in 2020 to 89 (53%) in 2023, 113 (67%) in 2025, to 155 (92%) by 2030 [6].

In addition, the 2030 agenda for Sustainable Development, which was accepted by the United Nations in 2015, details a blueprint for global targets to be achieved which will benefit both people and secure the health of the planet. These global targets are documented in the 17 Sustainable Development Goals (SDGs), developed in global partnership [7]. Ensuring health and promoting well-being is noted by the United Nations as being essential to sustainable development and intrinsically linked to poverty, captured under SDG 1 and 3. The SDG declaration states that in order to meet SDG 1 and 3, poverty everywhere must end, and universal health coverage and access to quality health care must be achieved. We used the WHO definition to describe universal health coverage as being health care that is accessible to all communities and people that require it, without resulting in financial hardship [8]. Rabies is acknowledged as a disease of poverty, and therefore ending poverty, and improving health and economic deprivation, could in theory result in a reduction of rabies, providing it also results in universal health coverage [4–6].

Development can be measured by different metrics, which vary in the extent to which they capture health coverage. Such metrics include total Gross Domestic Product (GDP), the percentage of total GDP spent on health care, and a metric of poverty assessing the extent and intensity of deprivation experienced within a population, the Multidimensional Poverty Index (MPI). Monitoring where improvement to health care access can occur, and therefore, where progress must be concentrated, can support the achievement of universal health coverage.

Gross Domestic Product (GDP) is the final value of goods and services produced within the geographic boundaries of a country during a specified time period, normally a year, and subsequently, the income earned from that production. GDP growth rate has commonly been used as an important indicator of the economic performance of a country [9]. How much a country spends on health care in relation to all other goods and services in the economy, and how these change over time is referred to as the current health expenditure (% of GDP). This provides a good indicator of how important health access is within the country relative to the whole economy. However, different countries use different definitions of GDP and so there is no global standardisation when calculating GDP.

An additional indicator that assesses poverty at the individual level is the global multidimensional poverty index (MPI). MPI was developed by the Oxford Poverty and Human Development Initiative (OPHI) with the UN Development Programme (UNDP) for inclusion in UNDP’s flagship Human Development Report in 2010 and is published annually [10]. The MPI is an international measure of multidimensional poverty covering over 100 lower middle income countries (LMICs, defined as countries with a Gross National Income (GNI) per capita between $1,046 and $4,095) [11],[12]. The countries included in the analysis make up three quarters of the world’s population with 22% identified as multidimensionally poor. It complements existing economic-based poverty measures such as GDP (current health care expenditure (%)) by capturing the deficiencies in health, education, and living standards and individual experiences at any given time. If a person is deprived in a third or more of the ten defined (weighted) indicators, the global MPI identifies them as ‘MPI poor’. Additionally, the intensity of a person’s poverty is also measured each year via the percentage of deprivations that person experiences [13]. The World Health Organization (WHO), World Organization for Animal Health (WOAH), and the Food and Agriculture Organization (FAO) together established the goal of eliminating dog-mediated human rabies deaths by 2030 [14–16]. Despite this disease being 100% preventable via the administration of post exposure prophylaxis (PEP), those who suffer dog bites may not seek medical advice, due to cost and availability, and accessibility [17,18]. Unless NTDs such as rabies, receive adequate resources, funding and prioritisation, they will continue to be neglected, with rabies continuing to be associated with poverty. There is good theoretical and empirical evidence that use of safe and effective available rabies vaccines will lead to reduction of rabies, yet economic and logistic barriers remain. [19–21] We investigated whether rabies incidence is an inevitable consequence of poverty or whether measures of development other than economics are stronger predictors of countries that have successfully controlled the disease. To do this, we quantified the level of association between health care access, poverty, and human deaths as a result of rabies, by applying four separate metrics that are currently used to measure the “wealth of a country” (total GDP, current health expenditure (% GDP), and MPI), and assessed the interconnectedness between them.

## 2. Methods

### 2.1. Data sources

Most recently available data (2020) for total GDP (US$), current health expenditure (% GDP), and MPI were extracted from publicly available sources from World Bank [22], and Oxford Poverty and Human Development Initiative [13]. Data for per capita human death rate due to rabies per 100,000 population, and the probability of receiving post exposure prophylaxis (PEP) were extracted from Hampson 2015 [23]. As reported figures for rabies deaths are considered underestimates of the true burden [23,24] the deaths rates used are an estimate using a model framework incorporating multiple data sources.

Death rate is used to assess rabies impact and receiving PEP is used to assess access to health care. Countries without data for a particular metric were excluded from that analysis (see results and S1 Data).

### 2.2. Calculating GDP dimensions

Total GDP can be calculated through three separate approaches (i) by combining all monies spent on goods and services minus the value of imports; (ii) the monies earned through wages and profits, (iii) and the value of goods and services produced. Current health expenditure as a percentage of gross domestic product (GDP) indicates the societal priority which is given to health in monetary terms.

### 2.3. Calculating MPI dimensions

The global MPI, used because it includes individual measures of poverty, identifies a person as MPI poor if they are deprived in a third or more of ten, weighted indicators, in 105 countries, and relies on surveys generated from Demographic and Health Surveys (DHS) (51 countries), Multiple Indicator Cluster Surveys (MICS) (43 countries), DHSMICS surveys (2 countries), Pan Arab Project for Family Health (PAPFAM) (three countries), and national surveys from six countries (Table 1). The MPI is calculated by multiplying the incidence of poverty (H), by the average intensity (A) across the poor (Eq 1) [10,25].


H*A=MPI (1)

**Table 1:** Dimensions, Indicators, Deprivation Cut-offs, and Weights for global MPI (Adapted from Oxford Poverty and Human Development Initiative).

Dimensions of poverty	Indicator	Deprived if living in a household where	Weight	SDG Area
Health (⅓)	Nutrition	Any person under 70 years of age for whom there is nutritional information is undernourished	1/6	SDG #2: Zero hunger
	Child mortality	A child under 18 has died in the household in the five year period preceding the survey	1/6	SDG #3: Health and well-being
**Education (⅓)**	Years of schooling	No eligible household member has completed six years of schooling	1/6	SDG #4: Quality education
	School attendance	Any school aged child is not attending school up to the age at which they would complete class 8	1/6	SDG #4: Quality education
Living standards (⅓)	Cooking fuel	a household cooks using solid fuel (dung, crops, shrub, wood, charcoal)	1/18	SDG #7: Affordable and clean energy
	Drinking water	The household’s source of drinking water is not safe or safe drinking water is 30-minutes or longer walk from home, roundtrip	1/18	SDG #6: Clean water and sanitation
	Sanitation	The household has unimproved or no sanitation facility or it is improved but shared with other household	1/18	SDG #6: Clean water and sanitation
	Electricity	The household has no electricity	1/18	SDG #7: Affordable and clean energy
	Housing	The household has inadequate housing materials in any of the three components: floor, roof, walls	1/18	SDG #11: Sustainable cities and communities
	Assets	The household does not own more than one of these assets: radio, TV, telephone, computer, animal cart, bicycle, motorbike, or refrigerator and does not own a car or truck	1/18	SDG #1: No poverty

### 2.4. Model Development

Data were fitted to generalised linear regression models. Pearson’s correlation coefficient (PCC) was used as a measure of linear correlation for the linear models (Eq 2). All models were developed in RStudio version 3.6.0 and are detailed in S1 Table. We also conducted additional analyses specifically on the subset of regions where higher endemicity is consistently reported, therefore for the purpose of these analyses we assumed a per capita death rate due to rabies above 0.6 per 100,000 population. Countries which were not included in the analyses due to unavailable data are listed in S1 Data.


$$r=\frac{\Sigma (xi-\bar{x})(yi-\bar{y})}{\sqrt{\Sigma (xi-\bar{x})2\Sigma (yi-\bar{y})2}}$$ (2)

Where:

r = correlation coefficient

xi = values of the x-variable in a sample

$\bar{x}$ = mean of the values of the x-variable

yi = values of the y-variable in a sample

$\bar{y}$ = mean of the values of the y-variable

## 3. Results

### 3.1. Total Gross Domestic Product (current US$) and per capita death rate from rabies

**Fig 1:**
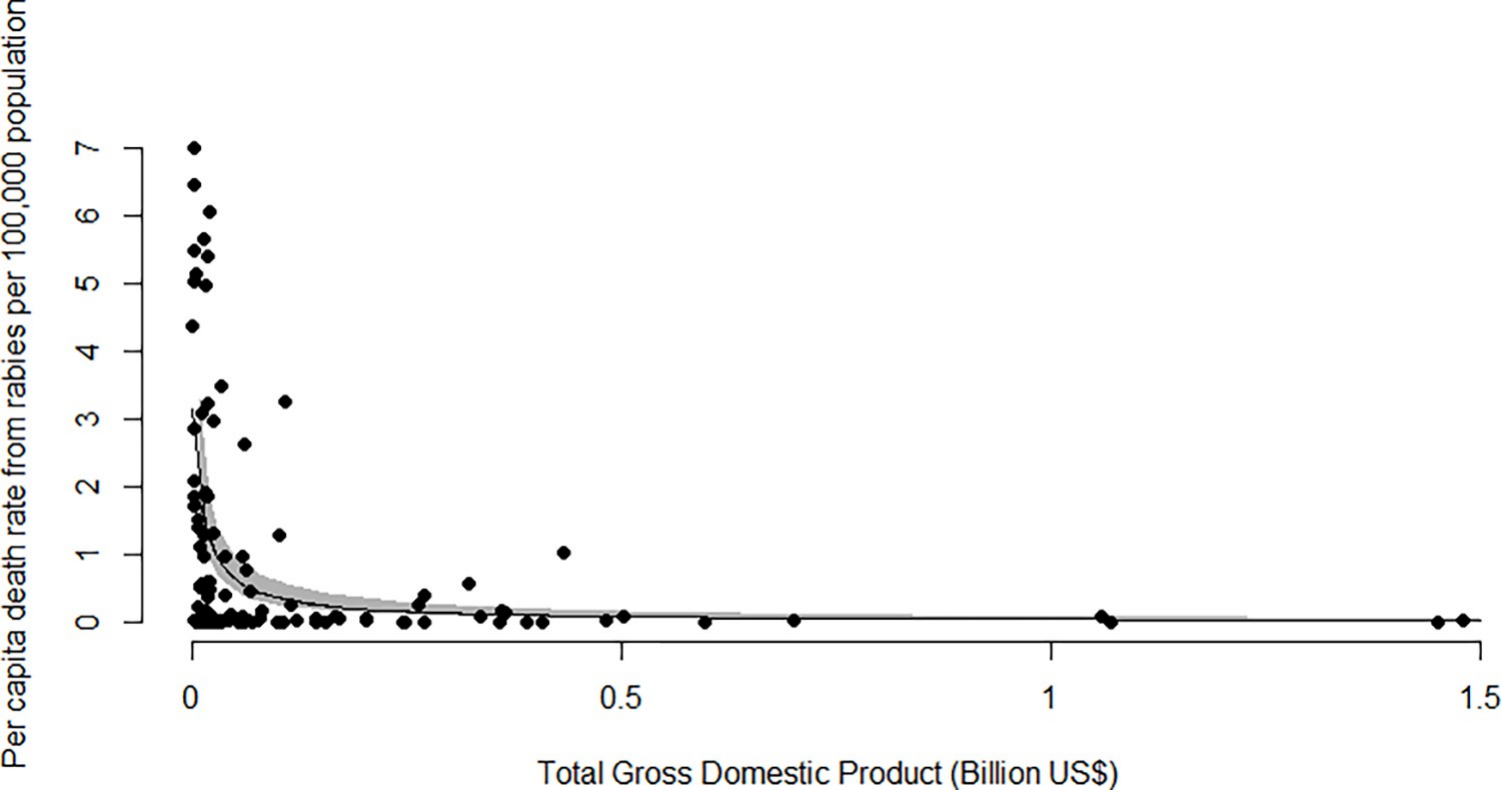
Results from the Generalised Linear Regression Model with Gamma distribution showing the relationship between total Gross Domestic Product (current US$) from 2020, and per capita death rate from rabies per 100,000 population from 2015, for 112 countries where data were available. (Minus outliers Myanmar, India, China, Turkey).

To explore if economically wealthier countries report a lower death rate from rabies, 112 countries reporting per capita death rate from rabies and total GDP were analysed. No statistically significant relationship was found between per capita death rate per 100,000 population and total gross domestic product (current US$) (p-value = 0.146711) (Fig 1).

### 3.2. Current health expenditure (% GDP) and per capita death rate from rabies per 100,000 population

**Fig 2:**
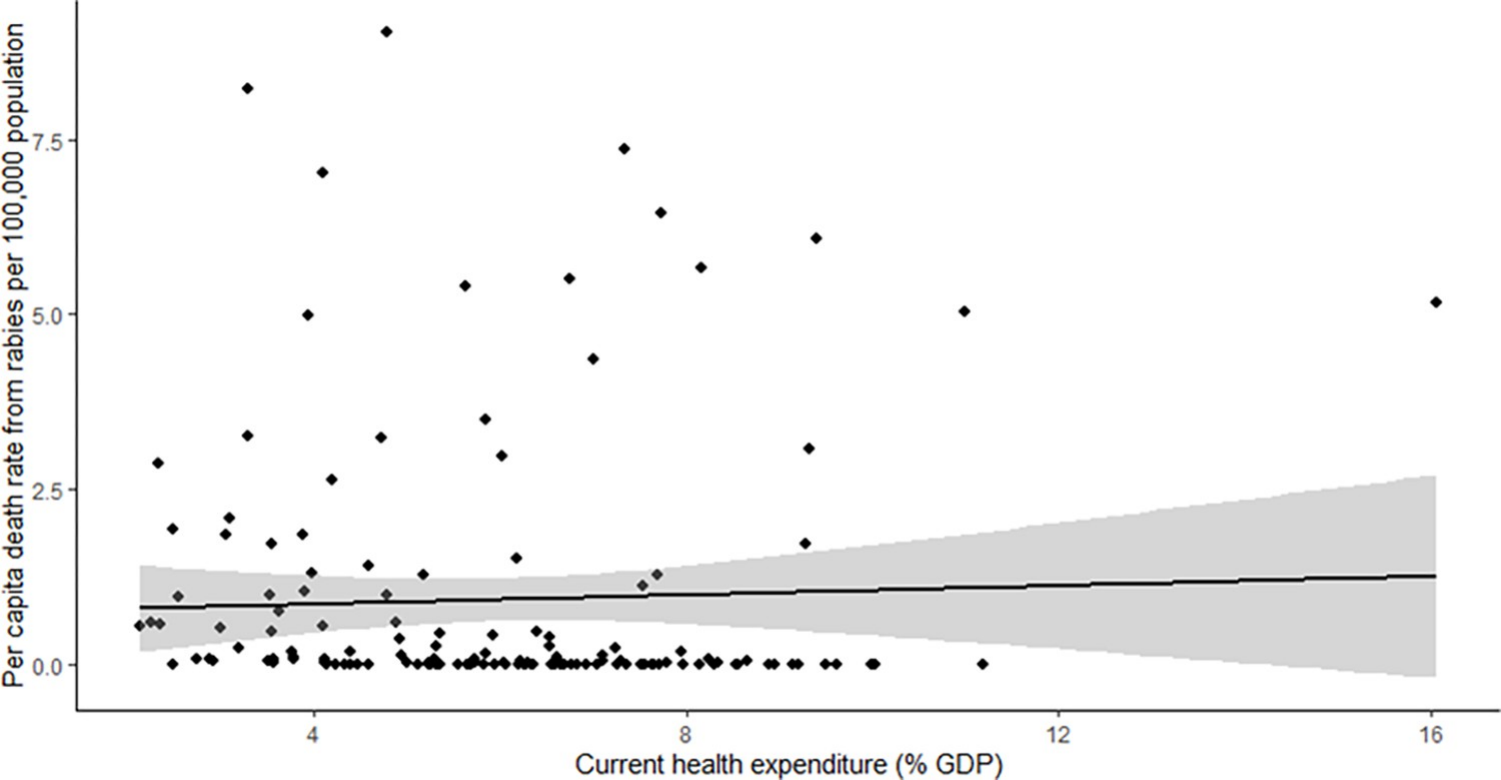
Results from Linear Regression with Poisson distribution, showing the relationship between current health expenditure (% GDP) from 2020, and per capita death rate from rabies per 100,000 population from 2015 for 146 countries where data were available.

Per capita death rate per 100,000 population for 146 countries were analysed for correlation with current health expenditure (% GDP). Pearson correlation demonstrated a weak association and non-significant association. (p-value = 0.64) (Fig 2). Analysis of a subset of countries reporting more than 0.6 deaths as a result of rabies, per 100,000 population also showed no significant association between health expenditure and rabies incidence (R2 = 0.081, p-value = 0.08) (S1 Fig).

### 3.3. Multidimensional Poverty Index and per capita death rate from rabies (100,000 population)

**Fig 3:**
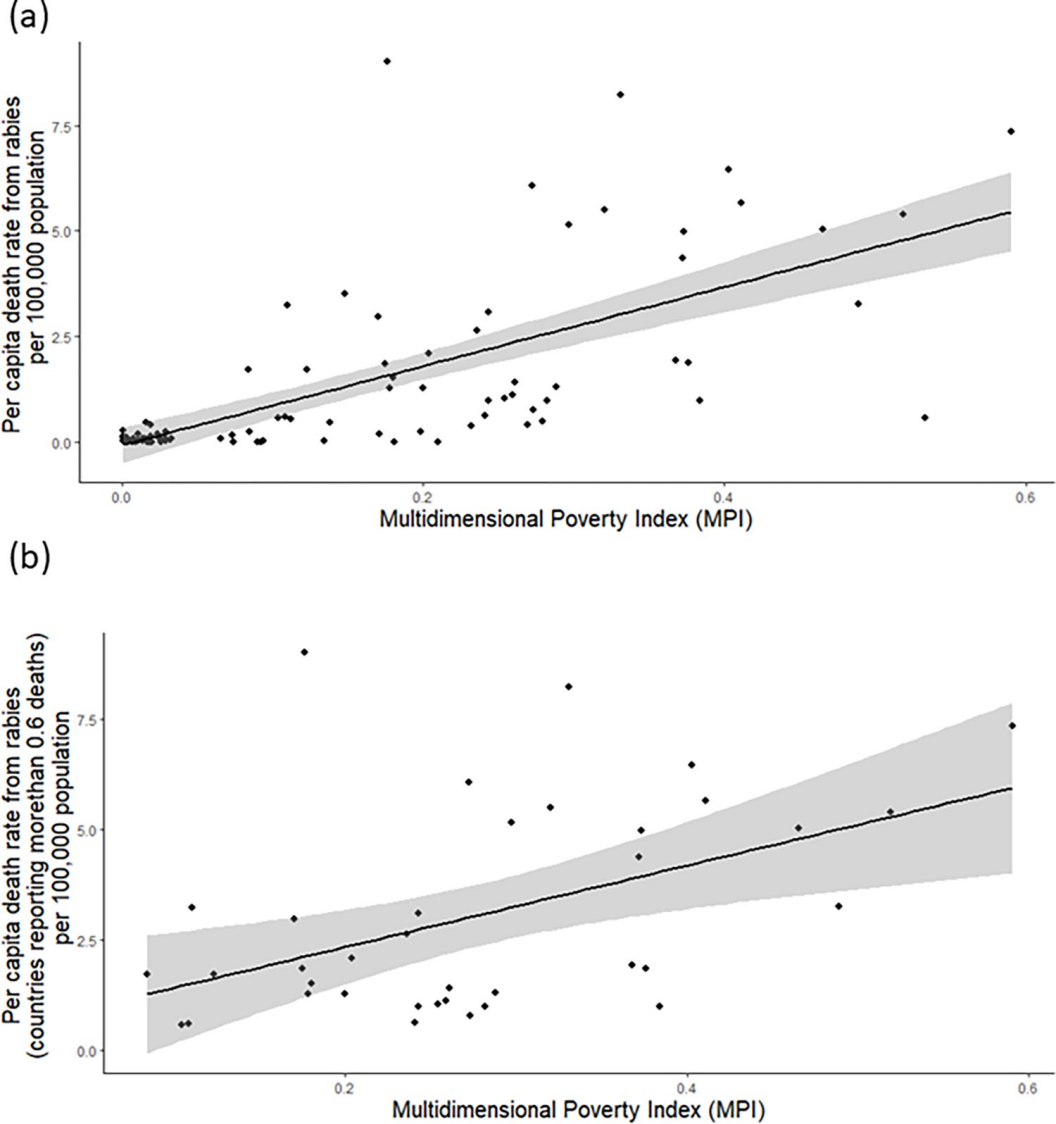
**(a)** Results from Linear Regression using Poisson distribution, between the two variables; Multidimensional Poverty Index from 2020, and per capita death rate from rabies per 100,000 population from 2015 for 98 countries where data were available. **(b)** Results from Linear Regression using Poisson distribution between the two variables; Multidimensional Poverty Index from 2020, and per capita death rate from rabies (countries reporting more than 0.6 deaths) per 100,000 population from 2015 for 37 countries where data were available.

Per capita death rate per 100,000 population was compared to MPI values reported for 98 countries (37 countries reporting more than 0.6 deaths as a result of rabies, per 100,000 population). We found that the higher the MPI ranking within countries, the higher the death rate from rabies, with a statistically significant relationship p-value = 6.4e-15 (95% CI (0.5645110–0.7785213) (Fig 3A); countries reporting more than 0.6 rabies deaths p-value = 0.0027 (95% CI (0.1835169–0.6951647) (Fig 3B).

### 3.4. Probability of a bite victim receiving PEP

**Fig 4:**
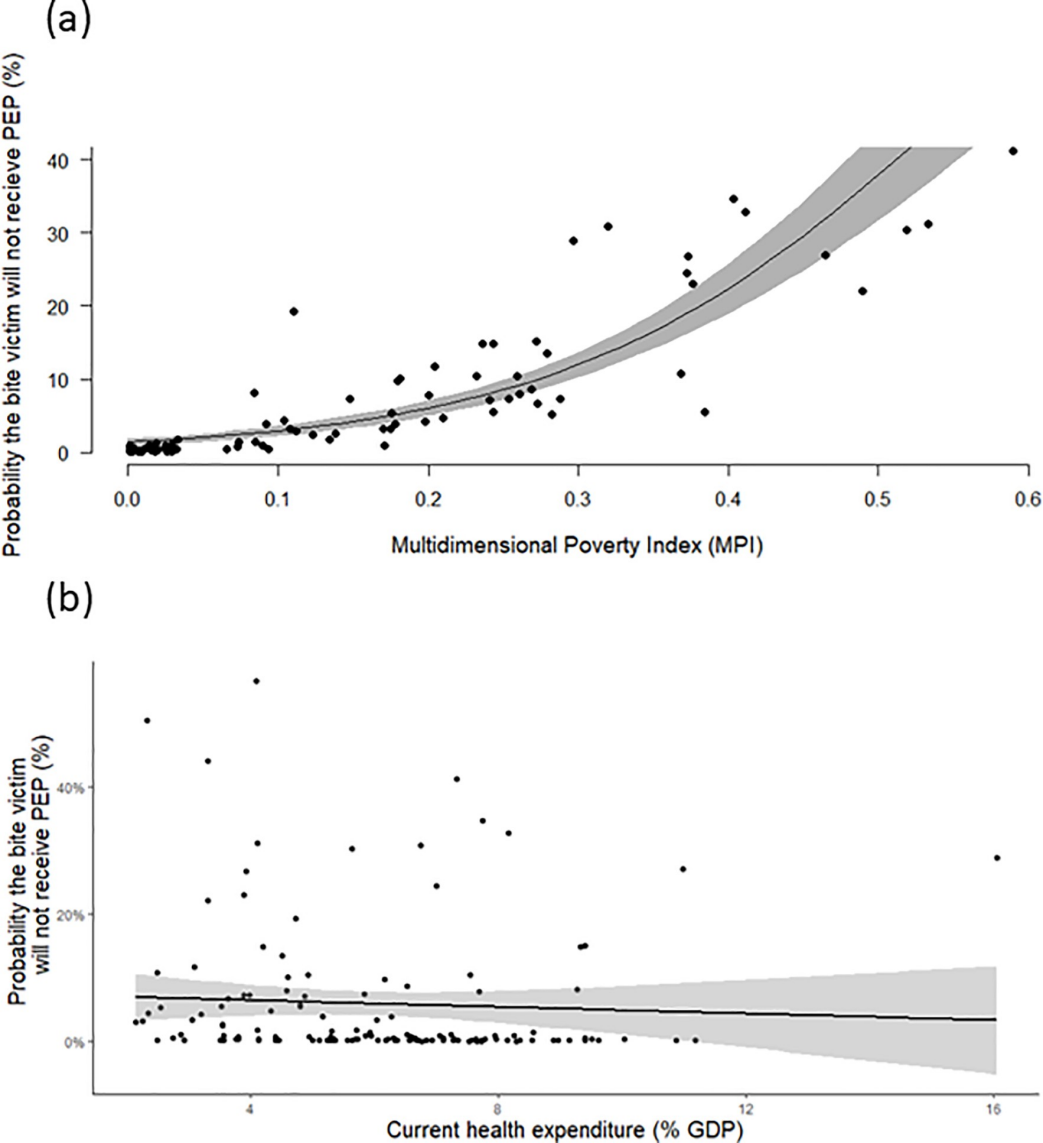
**(a)** Results from Generalised Linear Model using Quasibinomial distribution for current health expenditure (% GDP) from 2020, and probability of bite victims receiving PEP from 2015, in 98 countries where data were available. **(b)** Results from Generalised Linear Model for Multidimensional Poverty Index from 2020, and probability of bite victims not receiving PEP from 2015, in 98 countries where data were available.

Across the 98 countries examined, a higher MPI, that is, the poorer the average individual in the population, the higher the probability that a bite victim would not receive PEP, with a statistically significant relationship (p-value = 0.003776) (Fig 4A). However, across 137 countries no association was found to occur between current health expenditure (% GDP) and the probability of receiving PEP (p-value 0.535), again indicating that economic growth alone may not be sufficient in ensuring universal health access (Fig 4B).

Full summary of results can be found in S2 Table

## 4. Discussion

NTDs can act as indicators for the health status of communities, and also act as benchmarks for disparities in health care access [26]. By exploring three separate metrics associated with economic development, wealth, and poverty, we demonstrate that countries experiencing higher levels of poverty also report a higher human death rate from dog rabies, as well as poorer health access, when compared to wealthier countries. However, the results provide clear evidence that economic growth, and investment in health care, alone (as measured by GDP and expenditure on healthcare), is not useful at predicting the health status of communities and will not be enough to meet the global goal of eliminating dog mediated human rabies deaths by 2030.

Further, additional interventions applicable to rabies such as investment in control in the reservoir; dog vaccination, responsible pet ownership and community engagement, and free roaming dog management will also be needed. However, even with adequate resources and sufficient economic investment, without political will and commitment from decision makers, prioritisation and inclusion of disease interventions in long term national budgets, maintaining sustainable and effective control may be challenging.

Findings such as these, support the creation of tools such as the Global Dog Rabies Elimination Pathway (GDREP) to support public health officials plan the appropriate resources needed to achieve rabies elimination by 2030 through mass vaccination of dogs.

No significant relationship was found between current health expenditure and the probability of not receiving PEP, meaning that the proportion of GDP spent on health care did not influence the ability of at risk populations to access it. Conversely, a statistically significant relationship was found to be associated with development and the probability of a bite victim being able to access PEP. GDP has several limitations; for example, a monetary measure only measures market transactions, additionally quality is not calculated, and distribution of goods, and infrastructure are not included. GDP does not provide information on income distribution across countries or communities and therefore cannot highlight disparities which may be occurring [27]. Additionally, even if GDP per capita increases, the average person may not be in a better position financially, which implies unequal distribution of income and wealth, and therefore unequal opportunities for people across communities. Consumption expenditure as a measure of individual wellbeing can be problematic, as valuable indicators capturing the quality of health or the value of knowledge, cannot be evidenced. Human deprivation is multidimensional, reducing deprivation across one dimension may not automatically mean that reduction occurs across another dimension. Lastly, while GDP is an important indicator which captures the economic growth of a country, it fails to provide evidence of people’s well-being for which other available indicators may be more appropriate. Despite finding a no significant relationship between per capita death rate per 100,000 population and total gross domestic product (current US$) it is important to note that the highest human deaths as a result of dog rabies are known to occur in the low GDP areas [28, 29].

GDP measures economic growth within countries, however this indicator fails to capture the level of poverty experienced at the individual level, by each person. One person’s experience in one community may be dissimilar to another. MPI indicators capture this important information which provides evidence on the poverty experienced by individuals, and further can support the SDG 1 of ending poverty everywhere. We found the higher the MPI ranking within countries, that is the poorer an individual is, the higher the number of rabies cases. Importantly, we also found that the higher the country’s MPI ranking the lower the probability that the bite victims would receive PEP. This interconnectedness between MPI and rabies may be explained by measures used including education and living standards, that will influence crucial risk factors such as awareness of rabies and ability to travel to obtain PEP. MPI provides invaluable evidence to identify those most at risk thus, potentially reducing human suffering and further deprivations via targeted use of resources and amending policy. MPI data is not yet available to capture the impact that the COVID-19 pandemic has had on global poverty, but it is suggested that, if left unaddressed, progress towards achieving the SDGs may be delayed from between 3–10 years [30]. The COVID-19 crisis has also had a direct impact on progress made towards achieving rabies control; the availability of PEP and dog vaccinations was disrupted due to restrictions imposed on importations and distribution, and a change in free roaming dog movements, and demographics [31, 32]. There are limitations to the MPI; while this index highlights the disparities experienced within poor communities, it fails to capture all of these individual disparities. Therefore, the development of a tool to test the robustness of the rankings and parameters, such as poverty cut-offs, and deprivation cut-offs, used in the index is needed.

An important issue when assessing indices such as the ones included in this study, is the requirement of consistent data. Data on GDP, and MPI were unavailable from a number of countries and therefore had to be excluded from this study. We used most recently available data for each poverty and development metric (2020), however for epidemiological data (per capita death rate due to rabies per 100,000 population, and probability of receiving post exposure prophylaxis), the most complete data is from 2015 [23]. This clear limitation to our study highlights the need for reliable and dedicated regional rabies surveillance, and reporting systems to inform policy decisions. We note that it is reasonable to expect poverty and health care access to have improved between 2015 and 2020. A further potential confounding factor is that improved expenditure on healthcare may lead to improved surveillance and reporting, thereby increasing the reported rabies incidence. Data estimates used to determine the probability of receiving post exposure prophylaxis, is estimated only for those bite victims exposed to rabies virus and does not include all bite victims. Additionally, where these data were available for specific countries, they were used with Human Development Index (HDI) to extrapolate the probability of receiving PEP across other countries, where PEP data were available. It is therefore reasonable to expect that correlation between these two variables will occur. Data which allow for the assessment of burden of disease, and identification of those communities deemed most at risk of exposure is crucial in rabies control.

The Global Burden study predicts that globally, life expectancy overall could increase by 4.4 years between 2016 and 2040. But if less progress is made, life expectancy could decrease by 0.4 years for males and plateau for females [33]. The same study shows that people’s health can improve, but will depend on improving resources, and continued prioritization of health. Based on past trends, most countries’ Sustainable Development Goals (SDG) index scores were projected to rise between 2017 and 2030. However, despite this success, it is estimated that many SDG’s will fail to be achieved by 2030 unless progress based on health related indicators increases substantially between now and 2030. We highlight the important of drawing on different types of knowledge such as that generated from GDP and MPI indices, moving beyond single sector priorities so that the global goal of eliminating dog-mediated human rabies deaths by 2030 can be achieved. Other activities which come under SDGs, and which are related to NTD roadmap goals, and specifically rabies, are those conducted by other ministries or authorities. These include water and sanitation (WASH), and hygienic conditions for case management, for example wound washing associated with rabies exposure, agriculture, environment, livestock and wildlife, applicable to a one health approach for rabies control. Our results are not limited to improving only SDG 1 and 3, but also indirectly impact SDG 2 via the reduction of livestock and working animal losses to rabies, thus improving food safety. Additionally, with the involvement of local authorities to improve and manage dog control measures and responsible dog ownership, and waste management, allow progress towards achieving SDG 11 (sustainable cities and communities) and may lead to a reduction in spill over events to vulnerable or endangered wildlife populations which will contribute to environmental wellbeing, captured under SDG 15. Lastly, the forementioned activities, when combined, will build stronger relationship and help to sustain One Health partnerships, (SDG 17).

For this study, we focused on quantitative measurable indicators which capture the economic, and health status of a country, which as a result may reflect that country’s ability to achieve rabies elimination. However, there are many challenges both between countries, and within them. These include political will and stability, economic support, dog ownership attitudes and dog ecology capturing data on dog movement and behaviour. Adequate data on the disease burden within countries, which when coupled with disease dynamics models can be used to design locally specific vaccination campaigns that make best use of limited resources, however further analysis exploring the extent to which individual indicators making up the MPI are needed to explore which may have the greatest effect on rabies control. The study shows elimination of dog rabies in many countries with low development indices has been possible [34], so strategies used in the more developed countries should be adopted by less developed countries with a high incidence of rabies.

## 5. Conclusion

With increased development and availability of control measures, many countries have already eliminated dog rabies, demonstrating that it is possible to reach the goal of zero human deaths as a result of dog mediated rabies by 2030. However, economic growth alone may not be enough in many settings. Therefore, if we are to achieve SDG’s and the 2030 rabies elimination goals focus should be directed to cost-effective and sustainable control programmes such as mass dog vaccination using reliable real world data on dog demographics and movement. Indeed, other important development indications relevant to rabies control such as improving access to schooling, and ensuring universal health care access, particularly to the poorest communities, are also needed.

## Supporting information

S1 Table Summary of models used, indicating the response and explanatory values, and where data were omitted from analysis. (DOCX)

S2 Table Summary of the results, detailing sample size of countries include in each analysis, R^2^ / t-value, and p-value. (DOCX)

S1 Fig Results from Linear Regression model with Poisson distribution showing the relationship between current health expenditure (% GDP) from 2020, and per capita death rate from rabies per 100,000 population from 2015 (countries reporting more than 0.6 human deaths due to rabies). R2 = 0.081, p = 0.08.
(TIF)

S1 Data Data included per analysis. (DOCX)
