# Supplementary material for: Quantifying the interconnectedness between poverty, health access, and rabies mortality
Source: PLoS Negl Trop Dis. 2023 Apr 20;17(4):e0011204. doi: 10.1371/journal.pntd.0011204 (PMC10118163; doi:10.1371/journal.pntd.0011204)
Supplement: S1 Table — (DOCX) [file pntd.0011204.s001.docx]

| **Model Type** | **Response/dependant variable (y)** | **Explanatory/independent variable (x)** | **Data removed** | **Distribution** |
| --- | --- | --- | --- | --- |
| Linear Regression Model | Per capita death rate from rabies (100,000) | Gross Domestic Product (current health expenditure (%)) | those countries reporting a per capita death rate from rabies of more than .6 | Poisson |
| Generalised Linear Model | Per capita death rate from rabies (100,000) | Total Gross Domestic Product (current US$) | where no data were available | Gamma |
| Linear Regression Model | Per capita death rate from rabies (100,000) | Multidimensional Poverty Index | those countries reporting a per capita death rate from rabies of more than .6 | Poisson |
| Generalised Linear Model | Probability of receiving post-exposure prophylaxis (%) | Multidimensional Poverty Index | where no data were available | Quasibinomial |
| Generalised Linear Model | Probability of receiving post-exposure prophylaxis (%) | Current health expenditure (%GDP) | where no data were available | Quasibinomial |

S1 Table. Summary of models used, indicating the response and explanatory values, and where data were omitted from analysis.
