# Supplementary material for: Quantifying the interconnectedness between poverty, health access, and rabies mortality
Source: PLoS Negl Trop Dis. 2023 Apr 20;17(4):e0011204. doi: 10.1371/journal.pntd.0011204 (PMC10118163; doi:10.1371/journal.pntd.0011204)
Supplement: S2 Table — (DOCX) [file pntd.0011204.s002.docx]

S2 Table. Summary of the results, detailing sample size of countries include in each analysis, R^2^ / t-value, and p-value

| **Model Type** | **Response variable** | **Explanatory variable** | **Number of countries included** | **R^2^ / t-value** | **p-value** |
| --- | --- | --- | --- | --- | --- |
| Linear Regression Model | Per capita death rate from rabies (100,000) | Gross Domestic Product (current health expenditure (%)) | 145 | R^2^  0.0015 | 0.64 |
| Generalised Linear Model | Per capita death rate from rabies (100,000) | Total Gross Domestic Product (current US$) | 112 | t value  1.460 | 0.146711 |
| Linear Regression Model | Per capita death rate from rabies (100,000) | Multidimensional Poverty Index | 98 | R^2^  0.69 | 6.4e-15 |
| Generalised Linear Model | Probability of receiving post-exposure prophylaxis (%) | Multidimensional Poverty Index | 98 | z-score  2.897 | 0.003776 |
| Generalised Linear Model | Probability of receiving post-exposure prophylaxis (%) | Current health expenditure (%GDP) | 137 | t-value  -0.623 | 0.535 |
