# Supplementary figures and images for: Quantifying the interconnectedness between poverty, health access, and rabies mortality
Source: PLoS Negl Trop Dis. 2023 Apr 20;17(4):e0011204. doi: 10.1371/journal.pntd.0011204 (PMC10118163; doi:10.1371/journal.pntd.0011204)

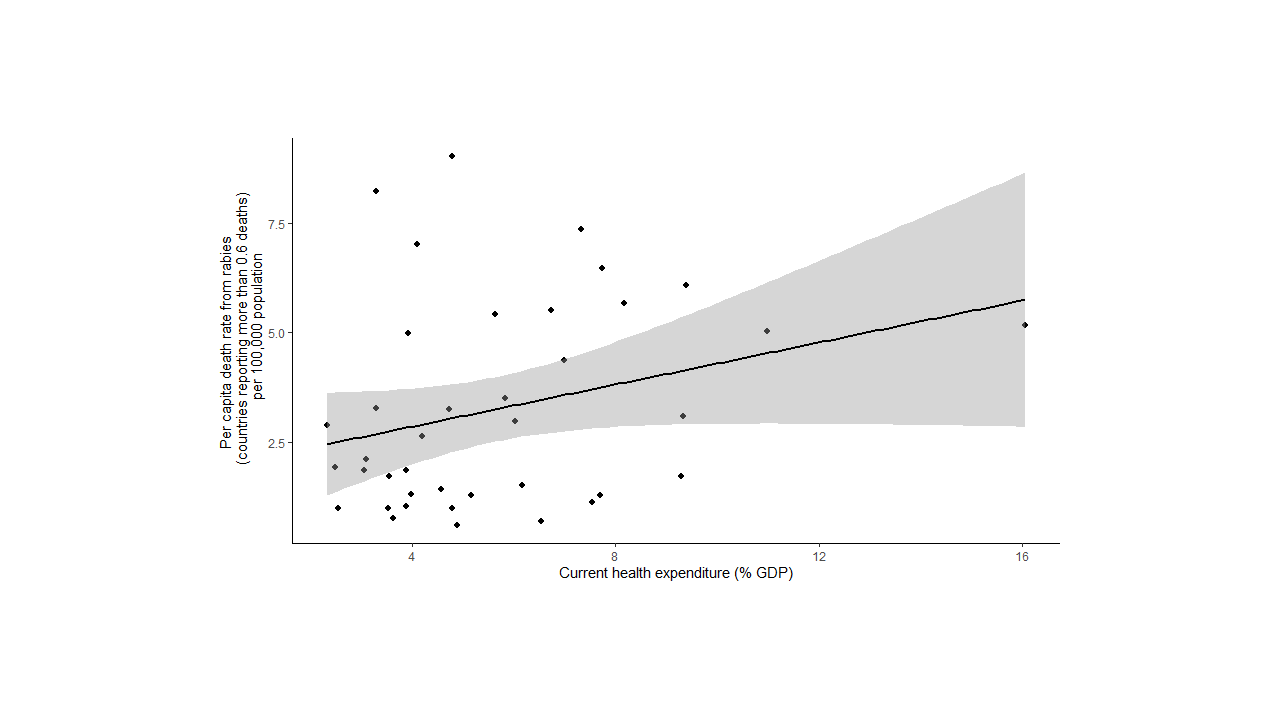

Supplement: S1 Fig — R2 = 0.081, p = 0.08. (TIF) [file pntd.0011204.s003.tif]
