# Supplementary material for: Quantifying the interconnectedness between poverty, health access, and rabies mortality
Source: PLoS Negl Trop Dis. 2023 Apr 20;17(4):e0011204. doi: 10.1371/journal.pntd.0011204 (PMC10118163; doi:10.1371/journal.pntd.0011204)
Supplement: S1 Data — (DOCX) [file pntd.0011204.s004.docx]

S1 Data

**Data included per analysis**

Total Gross Domestic Product (current US$) and per capita death rate from rabies per 100,000 population for 112 countries. No data were reported for (Democratic Republic of the Congo, Japan, Kyrgyzstan, New Zealand, Niger, Norway, Saint Kitts and Nevis, Saint Lucia, Saint Vincent and the Grenadines

Current health expenditure (% GDP) and per capita death rate per 100,000. No data were reported for, Japan, New Zealand, Niger, Norway.

Current health expenditure (% GDP) and countries reporting more than 0.6 human deaths due to rabies. No data were reported for Albania, Algeria, Antigua and Barbuda, Argentina, Armenia, Azerbaijan, Bahamas, Bahrain, Bangladesh, Barbados, Belarus Belize, Bolivia, Bosnia and Herzegovina, Botswana Brazil, Bulgaria, Cabo Verde, Chad, Chile, China Columbia, Comoros, Republic of Congo, Costa Rica, Croatia, Cuba, Cyprus, Czech Republic, Dominica, Dominican Republic, Ecuador, Egypt, El Salvador, Equatorial Guinea, Estonia, Gabon, Georgia, Ghana, Greece, Grenada, Guatemala, Guyana, Honduras, Hungary, Hungary, Indonesia, Iran, Iraq, Israel, Jamaica, Japan, Jordan, Kazakhstan, Kuwait, Kyrgyzstan, Kuwait, Kyrgyzstan, Lao Peoples, Democratic Republic, Latvia, Lebanon, Libyan Arab Jamahiriy, Lithuania, Malta, Mauritius Mexico, Moldova, Mongolia, Morocco, Namibia, New Zealand, Nicaragua, Niger, North Macedonia, Norway, Oman, Pakistan, Panama Paraguay, Peru, Philippines, Poland, Qatar, Romania, Russian Federation, Saint Kitts and Nevis, Saint Lucia, Saint Vincent and the Grenadines, Sao Tome and Principe, Saudi Arabia, Serbia, Seychelles, Slovakia, Slovenia, South Africa, Sri Lanka, Sudan, Suriname, Syrian Arab Republic, Tajikistan, Thailand, Timor-Leste, Trinidad and Tobago, Tunisia Turkey, Uganda, Ukraine, United Arab Emirates, United States of America, Uruguay, Venezuela, Vietnam, Zambia.

Multidimensional Poverty Index and per capita death rate from rabies per 100,000 population. No data were reported for Serbia, Palestine, Montenegro, Macedonia, Kiribati, eSwatini, Papa New Guinea , south Sudan, Antigua and Barbuda, Argentina, Australia, Austria, Azerbaijan, Bahamas, Bahrain, Belarus, Belgium, Brunei Darussalam, Bulgaria, Canada, Cape Verde, Chile, Costa Rica, Croatia, Cyprus, Czech Rep, Denmark, Djibouti, Dominica, Equatorial Guinea, Eritrea, Estonia, Finland, France, Germany, Greece, Grenada, Hungary, Iran, Israel, Italy, Japan, Kuwait, Latvia, Lebanon, Lithuania, Luxemburg, Malta, Mauritius, Mongolia, Netherlands, New Zealand, Norway, Oman, Panama, Poland, Portugal Qatar, Romania, Russian Federation, Saint Kitts and Nevis, Saint Vincent and the Grenadines, Saudi Arabia, Singapore, Slovakia, Slovenia, Spain, Sweden, Switzerland, Turkey, United Arab Emirates, UK, USA, Uruguay, Uzbekistan, Venezuela

Multidimensional Poverty Index and countries reporting more than 0.6 human deaths due to rabies. No data were reported for Albania, Algeria, Armenia, Barbados, Belize, Bolivia, Bosnia and Herzegovina, Botswana, Brazil, Chad, China, Columbia, Comoros, Congo, Cuba, Dominican Republic, Ecuador, Egypt, El Salvador, Gabon, Georgia Ghana, Guatemala, Guyana, Honduras, Indonesia, Iraq, Jamaica, Jordan, Kazakhstan, Kyrgyzstan, Libya, Maldives, Mexico, Moldova, Morocco, Namibia, Nepal, Nicaragua, Pakistan, Paraguay, Peru, Philippines, Saint Lucia, Sao Tome and Principe Seychelles, South Africa, Sri Lanka, Sudan, Suriname, Syrian Arab Republic, Tajikistan, Thailand, Timor-Leste, Trinidad and Tobago, Tunisia, Turkmenistan, Uganda, Ukraine, Vietnam, Zambia.

Multidimensional Poverty Index and the probability of a bite victim receiving PEP. No data were reported for Cape Verde, Djibouti, Equatorial Guinea, Eritrea, Mauritius, Somalia, Swaziland, Antigua and Barbuda, Argentina, Bahamas, Canada, Chile, Costa Rica, Dominican, Grenada, Panama, Saint Kitts and Nevis, Saint Vincent and the Grenadines, United States of America, Uruguay, Venezuela, Bahrain, Brunei Darussalam, Democratic Peoples of Korea, Iran, Israel, Japan, Kuwait, Lebanon, Malaysia, Mongolia, Oman, Qatar, Republic of Korea, Russian Federation, Saudi Arabia, Singapore, Turkey, United Arab Emirates, Uzbekistan, Andorra, Austria, Azerbaijan, Belarus, Belgium, Bulgaria, Croatia, Cyprus, Czech Republic, Denmark, Estonia, Finland, France, Germany, Greece, Hungary, Iceland, Ireland, Italy, Latvia, Lithuania, Luxemburg, Malta, Monaco, Netherlands, Norway, Poland Portugal, Romania, San Marino, Serbia and Montenegro, Slovakia, Slovenia, Spain, Sweden, Switzerland, The former Yugoslav Republic of Macedonia, United Kingdom, Australia, Cook Islands, Fiji, Kiribati, Marshall Islands, Micronesia (Federated States of), Nauru, New Zealand, Niue, Palau, Papua New Guinea, Samoa, Solomon Islands, Tonga, Tuvalu, Vanuatu.

Generalised Linear Model for current health expenditure (% GDP) and probability of bite victims not receiving PEP. No data were reported for Norway, Switzerland, Ireland, Hong Kong, Germany, Iceland, Australia, Sweden, Singapore, Netherlands, Denmark, Finland, Canada, New Zealand, United Kingdom, Belgium, Lichtenstein, Japan, Austria, Luxemburg, S. Korea, Spain, France, Italy, Andorra, Portugal, Brunei, Oman, Uruguay, Mauritius, Antigua and Barbuda, North Macedonia, Fiji, Tonga, Marshall Islands, Palestine, Micronesia, Eswatini, Vanuatu, Solomon Islands, Papua New Guinea, Ivory Coast, South Sudan. Myanmar, India, China, Turkey removed as outliers
